# A BDNF loop-domain mimetic acutely reverses spontaneous apneas and respiratory abnormalities during behavioral arousal in a mouse model of Rett syndrome

**DOI:** 10.1242/dmm.016030

**Published:** 2014-09

**Authors:** Miriam Kron, Min Lang, Ian T. Adams, Michael Sceniak, Frank Longo, David M. Katz

**Affiliations:** 1 Department of Neurosciences, Case Western Reserve University School of Medicine, 10900 Euclid Avenue, Cleveland, OH 44106, USA; 2 Department of Neurology and Neurological Sciences, Stanford University School of Medicine, 300 Pasteur Drive, Stanford, CA 94305, USA

**Keywords:** *Mecp2*, Brain-derived neurotrophic factor (BDNF), Respiration, Brainstem, Arousal

## Abstract

Reduced levels of brain-derived neurotrophic factor (BDNF) are thought to contribute to the pathophysiology of Rett syndrome (RTT), a severe neurodevelopmental disorder caused by loss-of-function mutations in the gene encoding methyl-CpG-binding protein 2 (MeCP2). In *Mecp2* mutant mice, BDNF deficits have been associated with breathing abnormalities, a core feature of RTT, as well as with synaptic hyperexcitability within the brainstem respiratory network. Application of BDNF can reverse hyperexcitability in acute brainstem slices from *Mecp2*-null mice, suggesting that therapies targeting BDNF or its receptor, TrkB, could be effective at acute reversal of respiratory abnormalities in RTT. Therefore, we examined the ability of LM22A-4, a small-molecule BDNF loop-domain mimetic and TrkB partial agonist, to modulate synaptic excitability within respiratory cell groups in the brainstem nucleus tractus solitarius (nTS) and to acutely reverse abnormalities in breathing at rest and during behavioral arousal in *Mecp2* mutants. Patch-clamp recordings in *Mecp2*-null brainstem slices demonstrated that LM22A-4 decreases excitability at primary afferent synapses in the nTS by reducing the amplitude of evoked excitatory postsynaptic currents and the frequency of spontaneous and miniature excitatory postsynaptic currents. *In vivo*, acute treatment of *Mecp2*-null and -heterozygous mutants with LM22A-4 completely eliminated spontaneous apneas in resting animals, without sedation. Moreover, we demonstrate that respiratory dysregulation during behavioral arousal, a feature of human RTT, is also reversed in *Mecp2* mutants by acute treatment with LM22A-4. Together, these data support the hypothesis that reduced BDNF signaling and respiratory dysfunction in RTT are linked, and establish the proof-of-concept that treatment with a small-molecule structural mimetic of a BDNF loop domain and a TrkB partial agonist can acutely reverse abnormal breathing at rest and in response to behavioral arousal in symptomatic RTT mice.

## INTRODUCTION

Complex respiratory disturbances, including atypical respiratory pauses and apneas, are a prominent feature of Rett syndrome (RTT), severely impacting health and quality of life (Katz et al., 2009; Ramirez et al., 2013). The severity of breathing dysfunction in individuals with RTT is strongly influenced by behavioral state: respiratory phenotypes worsen when patients are agitated and improve when patients are relaxed or sleeping (Katz et al., 2009; Ramirez et al., 2013). Increasing evidence from mouse models suggests that respiratory dysfunction in RTT is associated with a more excited default state in the brainstem respiratory network, including in cell groups involved in respiratory pattern generation (preBӧtzinger complex) and modulation [nucleus Koelliker-Fuse, the nucleus locus coeruleus and the nucleus of the solitary tract (nTS)] (Stettner et al., 2007; Katz et al., 2009; Ramirez et al., 2013). Recent findings of respiratory hyperreflexia in *Mecp2* mutant mice (Roux et al., 2008; Voituron et al., 2009) are consistent with network hyperexcitability, particularly in the nTS, which is the principal relay for primary afferent inputs to respiratory reflex pathways. In particular, hyperexcitability within lateral subnuclei of the *Mecp2* mutant nTS, where pulmonary stretch receptors form the first synapse in the Hering-Breuer reflex (HBR) pathway (Kubin et al., 2006), would be expected to decrease the activation threshold for the inspiratory off-switch and thereby promote the generation of apneas in RTT. These data suggest that therapeutic strategies aimed at restoring normal sensory gating in nTS by reducing synaptic hyperexcitability might ameliorate abnormal breathing in *Mecp2* mutant mice.

Brain-derived neurotrophic factor (BDNF), which is in deficit in RTT (Li and Pozzo-Miller, 2014; Katz, 2014), normally modulates excitability at primary afferent synapses in the nTS by inhibiting post-synaptic responses to glutamatergic excitation (Balkowiec and Katz, 2002). Consistent with these findings, short-term exposure to exogenous BDNF reduces synaptic hyperexcitability in the nTS in brainstem slices prepared from *Mecp2*-null mice (Kline et al., 2010), raising the possibility that restoration of BDNF-TrkB signaling might acutely improve respiratory control in RTT. However, BDNF itself does not have favorable drug-like characteristics, i.e. limited half-life and poor blood-brain barrier penetration, thus motivating the search for alternative approaches to enhancing BDNF-TrkB signaling in RTT (Ogier et al., 2007; Deogracias et al., 2012; Johnson et al., 2012; Schmid et al., 2012). In the present study, we used *in vitro* electrophysiology and *in vivo* plethysmography to examine the acute effects of LM22A-4, a small-molecule non-peptide BDNF loop-domain mimetic (Massa et al., 2010), on synaptic hyperexcitability in respiratory subnuclei of the nTS, as well as on abnormal breathing at rest and during behavioral arousal in *Mecp2* mutant mice. We previously found that chronic daily administration of a low dose of LM22A-4 [50 mg/kg body weight, intraperitoneally (i.p.)] reverses deficits in TrkB signaling in the brainstem in *Mecp2* mutants and improves resting breathing frequency (Schmid et al., 2012). In the present study, we unexpectedly found that acute administration of LM22A-4 results in complete reversal of spontaneous apneas, a key feature of breathing dysfunction in RTT. In addition, the present study reveals a potential synaptic mechanism for this acute effect by demonstrating that LM22A-4 decreases synaptic excitability in brainstem respiratory nuclei in *Mecp2*-null mice. Moreover, we demonstrate that respiratory dysregulation during behavioral arousal, a feature of human RTT, is also reversed in *Mecp2* mutants by acute treatment with LM22A-4. Our results are consistent with the hypothesis that deficits in BDNF-TrkB signaling contribute to breathing abnormalities in RTT and support the possibility that BDNF loop-domain mimetics could be useful in the acute treatment of respiratory dysfunction, including dysregulated responses to behavioral arousal, in RTT patients.

**TRANSLATIONAL IMPACT**

**Clinical issue**

Rett syndrome (RTT) is a devastating neurological disorder that represents one of the most common genetic causes of severe physical and intellectual disability in women. It affects approximately 1 in 10,000 female births and is caused by loss-of-function mutations in the gene encoding methyl-CpG-binding protein 2 (MeCP2), a transcriptional regulatory protein. Loss of MeCP2 disrupts multiple neurological systems, inducing marked cognitive, motor, respiratory and autonomic dysfunction. Among the core symptoms of RTT, difficulties with control of breathing are particularly troubling; they include irregular breathing, characterized by alternating periods of apneas, breath-holds and hyperventilation, and worsen with behavioral arousal. These respiratory dysfunctions severely impact quality of life and no treatments are currently available.

**Results**

*Mecp2* mutant mice are a well-established model of human RTT. These mice have reduced levels of brain-derived neurotrophic factor (BDNF), and this alteration has been linked to the etiology of RTT-related respiratory dysfunction, including abnormal excitability in brainstem networks that regulate breathing. BDNF is a member of the neurotrophin family of neuronal growth factor proteins that, in addition to its trophic functions during early development, plays a crucial role in regulating synaptic strength and neuronal excitability throughout life. In the present study, the authors examined the ability of LM22A-4, a small-molecule BDNF loop-domain mimetic and TrkB partial agonist, to ameliorate synaptic dysfunction in brainstem respiratory circuits and improve respiratory behaviors in *Mecp2* mutant mice. The authors demonstrated that, in brainstem slices from *Mecp2* mutant mice, application of LM22A-4 significantly reduced neuronal hyperexcitability in respiratory reflex pathways. In addition, acute treatment with LM22A-4 reversed RTT-associated breathing abnormalities, including spontaneous apneas and abnormal respiratory responses to behavioral arousal.

**Implications and future directions**

This study supports the hypothesis that RTT-associated respiratory dysfunctions in *Mecp2* mutant mice are linked to acute deficits in BDNF-TrkB signaling and that these dysfunctions can be rescued by the use of a small-molecule BDNF loop-domain mimetic. Moreover, the study suggests the feasibility of improving neurological function in RTT by redressing imbalances in neuronal excitability in respiratory regulative pathways. These findings point to the therapeutic potential of partial TrkB agonists such as LM22A-4 for treating respiratory dysfunctions in RTT.

## RESULTS

### LM22A-4 reduces synaptic excitability in the lateral, ventrolateral and interstitial subnuclei of *Mecp2*-null mice

We previously showed that *Mecp2*-null mice exhibit synaptic hyperexcitability within respiratory cell groups in the nTS, including in the lateral, ventrolateral and interstitial subnuclei (referred to here collectively as lnTS). Therefore, we decided to use the lnTS as a model system to test the ability of LM22A-4 to reduce synaptic hyperexcitability in the brainstem respiratory network of *Mecp2*-null mice. Male null mice, rather than female heterozygotes, were used for these experiments because the heterozygotes are mosaic for wild-type (Wt) and *Mecp2* mutant cells, which cannot be distinguished in the living slice preparation.

### Synaptic hyperexcitability in the *Mecp2*-null lnTS

**Fig. 1.**
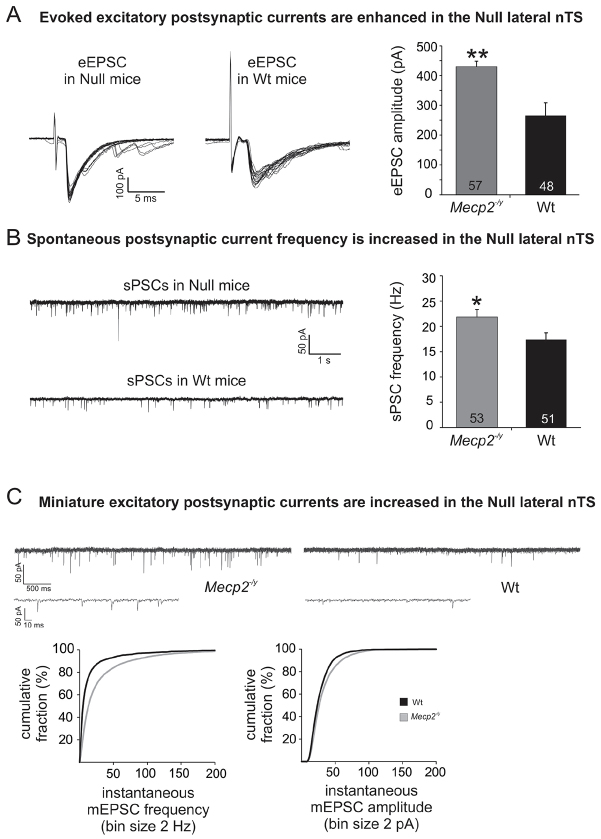
***Mecp2*-null mice exhibit synaptic hyperexcitability in lnTS.** (A,B) Raw traces and summary graphs illustrating increased eEPSC amplitude (A) and increased sPSC frequency (B) at primary afferent synapses in lnTS in *Mecp2*-null (Null) mice compared with wild-type (Wt) controls. *P≤0.05; **P≤0.01. In this and all subsequent graphs, the numbers within each bar indicate group size (*n*). (C) Raw traces and summary data illustrating increased mEPSC frequency and amplitude at primary afferent synapses in lnTS in *Mecp2*-null mice compared with Wt controls. Cumulative distribution curves of mEPSC frequency (left) and amplitude (right) demonstrate significant right shifts in Null mice (gray line) compared with Wt controls (black line) (*P*<0.05, Kolmogorov-Smirnov test).

Initial recordings of evoked, spontaneous and miniature excitatory postsynaptic currents (eEPSC, sPSC and mEPSC, respectively) from lnTS second-order neurons were performed to confirm and define in more detail the hyperexcitability phenotype previously observed in *Mecp2* nulls (Nulls) (Kline et al., 2010; Kron et al., 2012). Indeed, upon 0.5 Hz tractus solitarius (TS) stimulation, eEPSC amplitudes were significantly larger in the Null lnTS compared with Wt lnTS (representative trace in Fig. 1A; Wt, 266.0±18.1 pA, *n*=48; Null, 430.2±43.0 pA, *n*=57, *P*<0.01, unpaired Student’s *t*-test). Similarly, the instantaneous frequency of spontaneous synaptic currents was significantly higher in the Nulls, resulting in a strong trend towards a higher number of events within a 2-minute period as well (representative trace in Fig. 1B; sPSC frequency: Wt, 17.4±1.3 Hz, *n*=51; Null, 21.8±1.5 Hz, *n*=53; *P*<0.05; number of events: Wt, 599.8±64.5; Null, 772.6±89.5, *P*=0.07), with no significant effect of genotype on sPSC amplitudes. In addition, the frequency and amplitude distribution plots for mEPSCs in the presence of tetrodotoxin (TTX; 0.5 μM) and bicuculline (10 μM) showed significant right shifts in the Nulls as compared with Wt, indicating higher mEPSC frequency and amplitude (Null, *n*=13; Wt, *n*=11; Kolmogorov-Smirnov test, *P*<0.05; Fig. 1C). We did not detect genotype differences in membrane potential (*V*_m_) and membrane resistance (*R*_m_); however, capacitance (*C*_m_) was significantly reduced in the Nulls compared with Wt (*C*_m_: Wt, 34.5±1.8 pF; Null, 29.6±1.1, *P*<0.05).

### LM22A-4 acutely reduces synaptic hyperexcitability in the Null lnTS

**Fig. 2.**
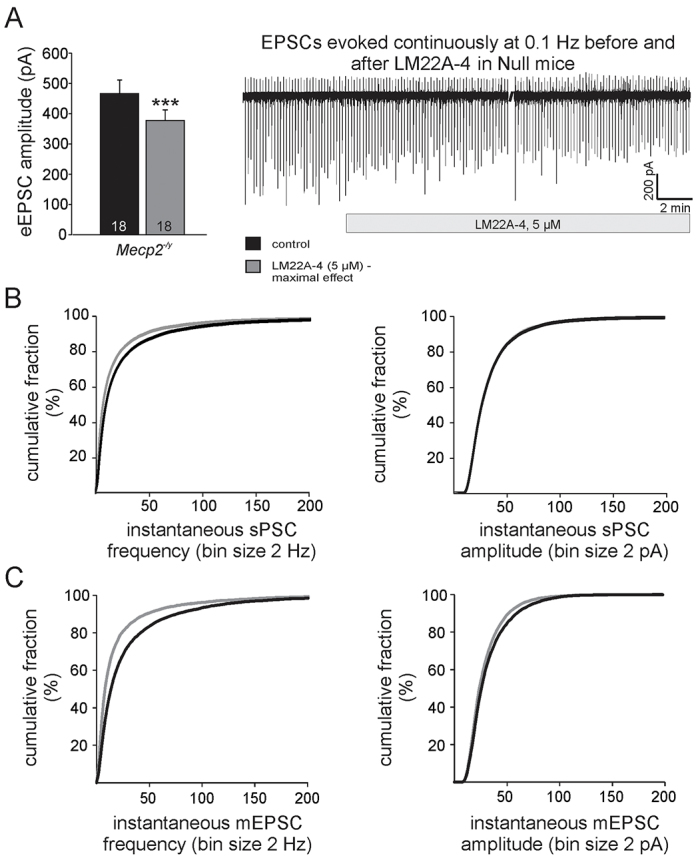
**LM22A-4 decreases synaptic hyperexcitability in the lnTS of *Mecp2*-null mice.** (A) The trace illustrates a long-term recording of EPSCs evoked by 0.1 Hz TS stimulation (downward deflections) in an lnTS neuron from a *Mecp2*-null (Null) mouse, and the reduction in eEPSC amplitude in response to LM22A-4. The accompanying graph compares maximal effects of LM22A-4 on eEPSCs in Null vs Wt lnTS neurons. Asterisks indicate significance of paired *t*-tests (control versus maximum effect); ***P<0.001. (B) Cumulative distribution plots of sPSC frequency (left) and amplitude (right) recorded in the absence (black line) and presence (gray line) of LM22A-4. The frequency plot demonstrates a significant left shift in the presence of the drug (*P*<0.05, Kolmogorov-Smirnov test). (C) Cumulative distribution plots of mEPSC frequency (left) and amplitude (right) recorded in the absence (black line) and presence (gray line) of LM22A-4 demonstrate significant left shifts in the presence of the drug (*P*<0.05, Kolmogorov-Smirnov test). See also supplementary material Fig. S1.

In slices from Null mice, bath-application of LM22A-4 (5 μM) significantly reduced eEPSC amplitudes in second-order lnTS neurons compared with pre-drug control recordings within 20 minutes (Fig. 2A; pre-drug control, 466.0±44.7 pA; LM22A-4, maximum effect, 377.4±35.0 pA, *n*=18, *P*<0.001, paired *t*-test). Specifically, eEPSC amplitudes decreased on average by 11.3±2.9% after 5–10 minutes of drug exposure [*P*<0.01 repeated measures (RM)-ANOVA], by 13.6±3.0% after 10–15 minutes, and by 17.1±4.0% after 15–20 minutes (*P*<0.001 RM-ANOVA). In addition, LM22A-4 caused a significant reduction in sPSC frequency as indicated by the left shift of the cumulative frequency distribution (Fig. 2B; *P*<0.05). As with eEPSC amplitudes, this effect was also time-dependent (average reduction in sPSC frequency by 21.8±4.5% after 8–10 minutes, by 24.6±3.1% after 13–15 minutes, and by 28.0±6.3% after 18–20 minutes of drug exposure; *P*<0.001 RM-ANOVA). Unlike eEPSC amplitudes, mean sPSC amplitudes were not affected by LM22A-4 (pre-drug control, 34.3±2.5 pA; LM22A-5, maximum effect, 33.7±2.7 pA, n.s.). In addition, LM22A-4 reduced both the frequency and, to a lesser degree, the amplitude of mEPSCs in the Nulls, reflected in left-shifts of frequency and amplitude distribution plots (*n*=13; Kolmogorov-Smirnov test, *P*<0.05; Fig. 2C). In contrast to Nulls, LM22A-4 had no significant effect on Wt eEPSC amplitudes and mean sPSC frequency and amplitude (supplementary material Fig. S1A; RM-ANOVA, n.s.). Analyses of mEPSC frequency and amplitude distribution curves in the presence and absence of LM22A-4 revealed virtually identical distributions in Wt cells (supplementary material Fig. S1B).

### LM22A-4 effects are blocked by K252a, a non-selective inhibitor of receptor tyrosine kinases

**Fig. 3.**
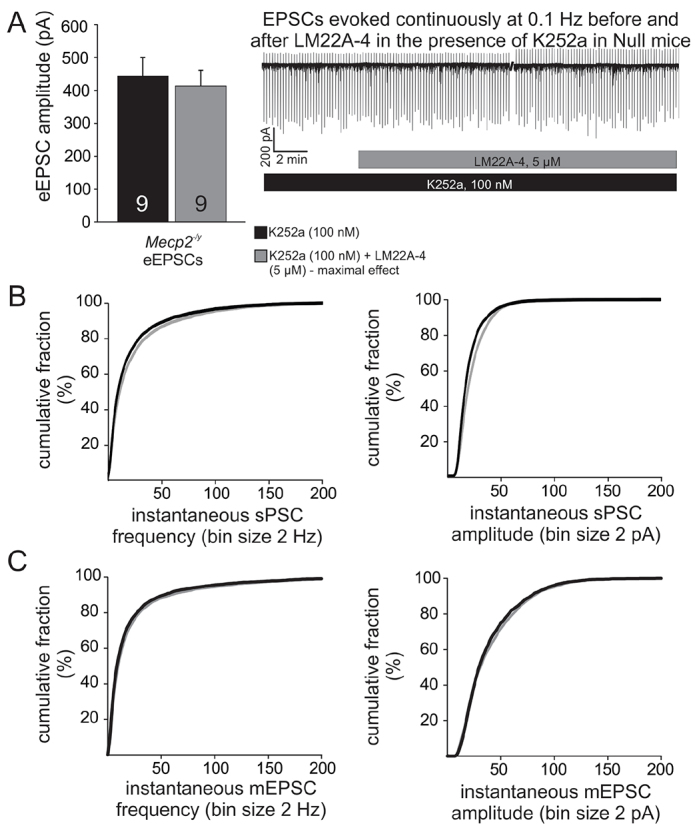
**LM22A-4 effects on synaptic hyperexcitability in *Mecp2*-null mice are inhibited by prior application of K252a.** (A) Raw trace and summary graph illustrate that, in the presence of the non-selective TrkB inhibitor K252a, LM22A-4 has no effect on eEPSC amplitude (*P*=0.23). (B,C) Cumulative distribution plots of sPSC (B) and mEPSC (C) frequency (left) and amplitude (right) recorded in the presence of K252a alone (black) versus K252a + LM22A-4 (gray) demonstrate virtually overlapping distributions despite small shifts in some regions that reach statistical significance (*P*<0.05, Kolmogorov-Smirnov tests).

Although LM22A-4 has previously been shown to selectively activate TrkB *in vitro* (Massa et al., 2010), this is the first study in which LM22A-4 has been applied to a slice preparation to test its effects on synaptic activity. Therefore, we next investigated whether or not prior application of K252a, a non-selective inhibitor of receptor tyrosine kinases, would reduce or eliminate the LM22A-4-mediated reduction of synaptic excitability in Nulls. Indeed, in the presence of K252a (100 nM), the effects of LM22A-4 on eEPSC amplitudes were abolished (Fig. 3A). Analyses of sPSC and mEPSC frequency distribution curves before and after LM22A-4 application in the presence of K252a revealed virtually overlapping distributions despite small shifts in some regions that reach statistical significance (Fig. 3B,C).

### LM22A-4 acutely reverses apneic breathing in *Mecp2* heterozygotes and nulls

**Fig. 4.**
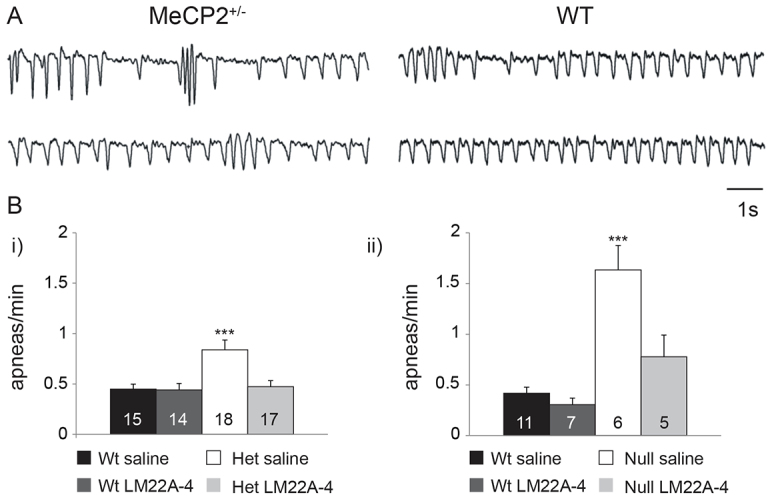
**Treatment with LM22A-4 *in vivo* acutely reverses the apnea phenotype in *Mecp2* heterozygotes and null mutants.** (A) Plethysmographic traces illustrating breathing patterns in saline-treated Het (left) and Wt (right) mice. (B) Summary graphs showing acute reversal of the apneic phenotype in 13- to 15-week-old Het (i) and 6- to 7-week-old Null (ii) mice following a single treatment with LM22A-4 (150 mg/kg body weight, i.p.) 1 hour prior to the beginning of breathing data collection. (Bi, ***P<0.001; Bii, ***P<0.001; ANOVA).

Our electrophysiology data demonstrated that, in *Mecp2*-null mice, LM22A-4 acutely reduces excitability at primary afferent synapses within the lnTS, a region that is crucial for regulation of the inspiratory off-switch (Kubin et al., 1985). Therefore, we next sought to determine whether or not acute treatment with LM22A-4 *in vivo* would ameliorate the apnea phenotype in *Mecp2*-null (Null) and -heterozygous (Het) mice; both genotypes exhibited qualitatively similar breathing phenotypes, which were more severe in the Nulls. For each gender, animals were divided into four groups, i.e. female Wt saline, *n*=15; female Wt LM22A-4, *n*=14; female Het saline, *n*=18; female Het LM22A-4, *n*=17 and male Wt saline, *n*=11; male Wt LM22A-4, *n*=7; male Null saline, *n*=6; male Null LM22A-4, *n*=5 (female mice were 13–15 weeks of age and male mice were 6–7 weeks of age). Analysis of quiet breathing in female Wt and Het mice revealed that saline-treated Hets exhibited approximately twice as many apneas per minute as saline-treated Wt animals (Fig. 4A,B). However, LM22A-4 treatment reversed the Het apnea phenotype, restoring the number of apneas to female Wt levels [Fig. 4Bi; apneas/minute: Wt saline, 0.45±0.05; Wt LM22A-4, 0.44±0.09; Het saline, 0.84±0.10; Het LM22A-4, 0.47±0.06, *P*<0.001 (*** in Fig. 4Bi) versus all other groups, ANOVA], even at doses as low as 5 mg/kg body weight (supplementary material Fig. S2). LM22A-4 had no effect on the number of apneas in Wt animals, and apnea length, respiratory frequency, inspiratory time, expiratory time and total breath duration were not affected by LM22A-4 treatment in animals of either genotype (not shown). Saline-treated male Nulls exhibited approximately four times as many apneas per minute as saline-treated Wt, and, as in Hets, LM22A-4 treatment restored the number of apneas to Wt levels [Fig. 4Bii; apneas/minute: Wt saline, 0.42±0.06; Wt LM22A-4, 0.30±0.07; Null saline, 1.63±0.24; Null LM22A-4, 0.78±0.22, *P*<0.001 (*** in Fig. 4Bii) versus all other groups, ANOVA]. To determine whether or not LM22A-4 treatment had prolonged effects on the apnea index, plethysmographic recordings were obtained from a subset of female Wt and Het mice 24 hours after LM22A-4 injection. These experiments demonstrated that the number of apneas per minute in LM22A-4-treated Hets had returned to the level of saline-treated Hets within 24 hours (apneas/minute: Wt saline, 0.56; Het saline, 1.15; Het LM22A-4, 1.52, *P*<0.128 versus all other groups, ANOVA; not shown). In contrast to previous reports from this and some other laboratories (Katz et al., 2012), the cohorts of Het mice used in the present study did not exhibit a respiratory frequency phenotype and no drug effect on resting frequency was observed.

### LM22A-4 acutely reverses abnormal respiratory responses to sensory arousal in *Mecp2* nulls

**Fig. 5.**
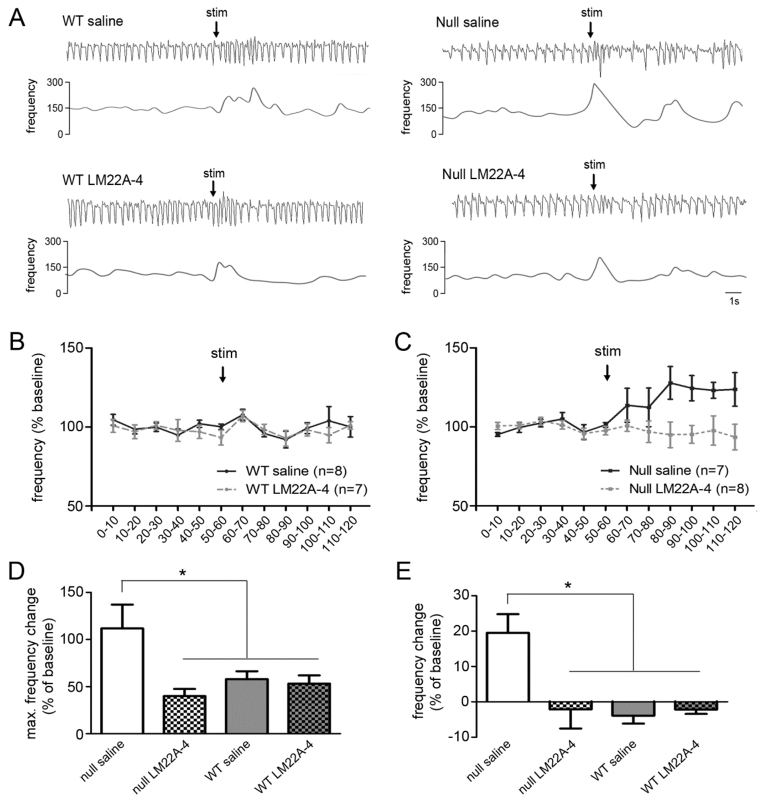
**LM22A-4 treatment acutely reverses abnormal respiratory responses to auditory arousal in *Mecp2*-null mice.** (A) Representative plethysmographic traces (top) with the corresponding instantaneous frequency plot from Wt and *Mecp2*-null (Null) mice treated with saline or LM22A-4 (150 mg/kg body weight, i.p.). stim, stimulation. (B,C) Mean respiratory frequency in 6- to 8-week-old saline- and LM22A-4-treated Wt (B) and *Mecp2*-null (C) mice recorded for 1 minute before and 1 minute after presentation of the auditory arousal stimulus (50 ms, 80 dB). LM22A-4 treatment abolished the abnormal increase in frequency seen in Nulls and had no effect in Wt mice. (D,E) Further analysis of the data shown in B and C, focusing on the peak of the transient frequency response recorded within 10 seconds after the auditory stimulus (D) as well as the sustained frequency response recorded during the full minute after the auditory stimulus (E). Treatment with LM22A-4 (150 mg/kg body weight, i.p.) reversed the transient (D) and prolonged (E) increases in mean respiratory frequency evoked by auditory stimulation in *Mecp2* nulls and had no effect in Wt animals. *P<0.05.

Because breathing abnormalities in RTT are strongly influenced by behavioral state (Ren et al., 2012; Ramirez et al., 2013), we next sought to determine whether or not LM22A-4 treatment would be effective at reversing abnormal state-dependent changes in breathing in *Mecp2* mutants. To approach this issue, we compared the respiratory component of the orienting reflex evoked by a brief auditory stimulus in saline- and LM22A-4-treated 6- to 8-week-old Wt and Null mice. The orienting reflex, which is evoked by novel sensory stimuli that are sub-threshold for evoking a startle reponse, is normally accompanied by a transient increase in respiratory frequency that returns to baseline within a few seconds (Nalivaiko et al., 2011). However, *Mecp2*-null mice exhibited an exaggerated respiratory component characterized by a significant increase in the amplitude of the transient response and the appearance of a secondary, sustained increase in frequency compared with Wt controls (Fig. 5) (M.S., M.L., D.M.K. et al., unpublished results). To test the hypothesis that this abnormal arousal phenotype is linked to reduced TrkB signaling, 6- to 8-week-old Wt and Null mice were treated with either saline or LM22A-4 (150 mg/kg body weight, i.p.) 1 hour prior to auditory stimulation. In Wt animals, we saw no effect of saline or drug treatment on the respiratory response to the auditory pulse. However, in Nulls, LM22A-4 treatment completely abolished the abnormal respiratory response to auditory stimulation, whereas saline injection had no effect [Fig. 5D: maximum frequency change within the initial 10 seconds following auditory stimulation: Null saline, 111.76±25.33; Null LM22A-4, 39.98±7.56; Wt saline, 57.89±8.38; Wt LM22A-4, 53.18±8.72, *P*<0.05 versus all other groups (* in Fig. 5D), ANOVA; Fig. 5E: average frequency change during 1 minute following auditory stimulation: Null saline, 19.5±5.24; Null LM22A-4, −2.10±5.46; Wt saline, −3.92±2.25; Wt LM22A-4, −2.19±1.25, *P*<0.001 versus all other groups (* in Fig. 5E), ANOVA].

### LM22A-4 treatment does not produce behavioral symptoms of sedation

**Fig. 6.**
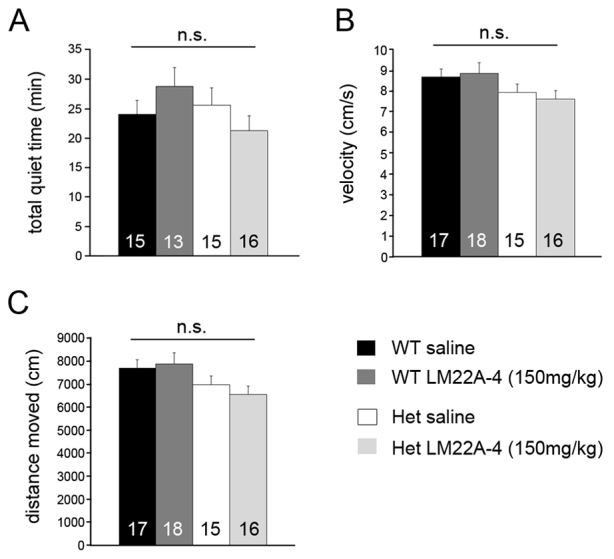
**LM22A-4 treatment has no significant effect on the total duration of quiet breathing time or open-field activity in Wt and Het mice.** Summary graphs comparing (A) the total amount of quiet breathing time (see Materials and Methods for details), (B) velocity of movement in an open field and (C) total distance traveled in an open field of 13- to 15-week-old Wt and Het mice 1–2 hours after injection with LM22A-4 (150 mg/kg body weight, i.p.).

Sedation has previously been shown to improve respiratory function in *Mecp2* mutants (Viemari et al., 2005; Ren et al., 2012). To determine whether or not LM22A-4 has sedating effects, we compared the total duration of quiet breathing periods per animal (see Materials and Methods) among saline- and LM22A-4 treated Wt and Het mice (13–15 weeks of age), using the same plethysmographic recordings used to generate the apnea dataset. This analysis found no difference in the duration of quiet breathing between saline- and drug-treated Hets (Fig. 6A). In addition, we compared open-field behaviors, which are often used to evaluate sedating drug effects (Broadhurst, 1960), in additional cohorts of Wt and Het mice treated with saline or LM22A-4 (150 mg/kg body weight, i.p.). Animals were tested 1–2 hours after injection, corresponding to the window of time during which breathing measurements were collected. These experiments revealed no effect of drug treatment on any behaviors in the open field, including velocity of movement (Fig. 6B) and the total distance traveled (Fig. 6C). Thus, by these measures, we were unable to detect evidence of sedation in LM22A-4-treated animals.

## DISCUSSION

The present results demonstrate the reversibility of synaptic and behavioral phenotypes associated with abnormal breathing in symptomatic *Mecp2* mutant mice, a model of RTT. Specifically, we found that LM22A-4, a BDNF loop-domain mimetic and TrkB partial agonist, reduces synaptic hyperexcitability within respiratory subnuclei in the nTS *in vitro*, and acutely eliminates apneic breathing and abnormal respiratory responses to sensory arousal following systemic administration *in vivo*.

### Acute reversal of apneas during resting ventilation

Our data support a link between reduced BDNF signaling in *Mecp2* mutants and the apneic breathing phenotype characteristic of RTT. Moreover, these data are consistent with previous results demonstrating that synaptic hyperexcitability in nTS is associated with reduced levels of BDNF (Wang et al., 2006; Ogier et al., 2007; Kline et al., 2010) and can be reversed by the application of exogenous BDNF in isolated brainstem slices (Kline et al., 2010). We hypothesize, therefore, that the ability of LM22A-4 to reduce or eliminate apneas in *Mecp2* mutants is directly related to its ability to reduce synaptic excitability in lnTS and thereby restore normal sensory gating of the inspiratory off-switch, possibly by enhancing input-output coupling in the HBR pathway (Dhingra et al., 2013). The present findings do not rule out the possibility that LM22A-4 might also act at sites other than the nTS to improve breathing in *Mecp2* mutants, including other BDNF-responsive cell populations in the brainstem respiratory network that regulate respiratory pattern generation, including the preBӧtzinger complex (Thoby-Brisson et al., 2003) and nucleus Kolliker-Fuse (Kron et al., 2007).

We found that hyperexcitability at primary afferent synapses in the lnTS in *Mecp2*-null mice is characterized by an increase in the frequency of spontaneous and miniature excitatory currents and an increase in the amplitude of currents evoked by primary afferent stimulation, suggesting both pre- and post-synaptic effects, respectively, of MeCP2 loss. Moreover, both of these synaptic phenotypes are reduced by application of LM22A-4. The reduction in eEPSC and mEPSC amplitudes following LM22A-4 treatment parallels previous results obtained with exogenous BDNF (Kline et al., 2010) and is consistent with a role for BDNF in modulating excitatory transmission at primary afferent synapses in the nTS by inhibiting postsynaptic AMPA receptors (Balkowiec et al., 2000). Although underlying mechanisms remain to be defined, a similar role for acute TrkB activation in inhibiting glutamatergic transmission has been described at excitatory synapses onto parvalbumin-positive cortical interneurons (Jiang et al., 2004). Given previous evidence that BDNF-TrkB signaling can positively or negatively regulate AMPA receptor trafficking to the plasma membrane (Rutherford et al., 1997; Narisawa-Saito et al., 2002; Caldeira et al., 2007; Li and Keifer, 2008; Keifer and Zheng, 2010; Reimers et al., 2014), reduced AMPA receptor availability might underlie the inhibitory effect of LM22A-4 on postsynaptic excitability in the mutant nTS. In addition, the reduction in sPSC frequency following LM22A-4 application indicates a presynaptic effect that is most likely mediated by an indirect pathway involving TrkB-expressing interneurons, because primary afferent inputs to the nTS do not express TrkB by 5 weeks of age (Kline et al., 2010).

### Acute reversal of abnormal respiratory responses to behavioral arousal

As we observed for apneic breathing in resting animals, acute treatment with LM22A-4 completely reverses the abnormal respiratory response to auditory arousal in *Mecp2* mutants. It is possible that both of these treatment effects result from reduced excitability within the respiratory network in mutants. However, it is also possible that LM22A-4 acts at mid- and/or forebrain sites that are important for behavioral state-dependent modulation of breathing, including the periaqueductal gray, amygdala and limbic cortices (Keay et al., 1988; Brandão et al., 1993; Nalivaiko et al., 2011).

In light of the fact that breathing dysfunction in RTT patients improves with relaxation or sleep (Weese-Mayer et al., 2008; Katz et al., 2009; Ren et al., 2012; Ramirez et al., 2013), we considered the possibility that, rather than, or in addition to, any direct effects on respiratory control per se, LM22A-4 might also improve breathing by inducing sedation. Indeed, other studies have shown that apneic breathing in RTT mouse models is reduced by drugs with sedating or anxiolytic effects, including the GABA uptake inhibitor NO-711 (Abdala et al., 2010), the GABA_A_ partial agonist L838,417 (Abdala et al., 2010), the 5-HT1A antagonist 8-OH-DPAT (Abdala et al., 2010), the benzodiazepines midazolam (Voituron and Hilaire, 2011) and diazepam (Abdala et al., 2010), the norepinephrine uptake inhibitor desipramine (Roux et al., 2007; Zanella et al., 2008), and the corticotropin-releasing hormone receptor 1 antagonist antalarmin (Ren et al., 2012). However, in the present study, open-field testing revealed no detectable differences in behaviors that are sensitive to sedation, including velocity of movement and total distance traveled, between saline- and drug-treated Hets. Similarly, drug-treated Hets showed no increase in the total duration of quiet breathing periods compared with their saline-treated counterparts. On the basis of these data, we conclude that sedation cannot explain the reversal of apneic breathing or normalization of the respiratory response to sensory arousal following treatment of *Mecp2* mutants with LM22A-4.

Other strategies for overcoming functional deficits associated with reduced BDNF-TrkB signaling in RTT include enhancing the levels of endogenous BDNF with ampakines (Ogier et al., 2007), which stimulate activity-dependent BDNF expression by enhancing AMPA receptor function (Lynch et al., 2008) or fingolimod (Deogracias et al., 2012), a sphingosine-1 phosphate receptor modulator. One potential limitation of these other approaches is that BDNF activates receptors other than TrkB, including p75. The properties of binding to p75 and/or functioning as a full TrkB ligand might play an important role in unwanted pleiotropic effects of elevated BDNF levels (Longo and Massa, 2013). LM22A-4, on the other hand, does not bind p75 and, in addition, does not fully compete for BDNF activation of TrkB (Massa et al., 2010). Thus, although LM22A-4 increases TrkB phosphorylation and activates downstream signaling pathways in multiple, but not all, models *in vivo* and *in vitro* (Kajiya et al., 2014; Massa et al., 2010; Han et al., 2012; Schmid et al., 2012; Todd et al., 2014), the range of actions of LM22A-4 is quantitatively and qualitatively distinct from that of BDNF (Massa et al., 2010). One attraction, therefore, of BDNF loop-domain mimetics functioning as TrkB partial agonists as potential RTT therapeutics might be the reduced risk of adverse effects that might otherwise be associated with excessive activation of p75 and TrkB. Currently, there are no treatments available for either resting breathing abnormalities or respiratory disruption during behavioral arousal in RTT, and our data raise the possibility that TrkB-targeted therapies could be effective in both contexts.

## MATERIALS AND METHODS

### Animals

*Mecp2^tm1.1Jae^* mice, developed by Dr R. Jaenisch (Whitehead Institute, Massachusetts Institute of Technology, Cambridge, MA) were purchased from the Mutant Mouse Regional Resource Center (University of California Davis, Davis, CA) and maintained on a mixed genetic background (129Sv, C57BL/6, BALB/c) by crossing *Mecp2^tm1.1Jae^* heterozygous females (*Mecp2^−/+^*; Het) with *Mecp2^tm1.1Jae^* wild-type males (Wt; *Mecp2^+/y^*) to generate *Mecp2^tm1.1Jae^*-null males (*Mecp2^−/y^*; Nulls) and *Mecp2^tm1.1Jae^* heterozygous females (*Mecp2^+/−^*; Hets). 6- to 8-week-old Nulls and 13- to 22-week-old Hets were used for experiments. All experimental procedures were approved by the Institutional Animal Care and Use Committee at Case Western Reserve University.

### Randomization and blinding

Prior to each experiment, animals were numerically coded and assigned to treatment groups by computerized random number selection. Drug and saline injections, plethysmography, open-field testing and data analysis were all performed by investigators blinded to genotype and treatment.

### Plethysmography

Breathing was recorded in unrestrained Wt, Het and Null mice using a whole-body plethysmograph (Buxco II; Buxco Research Systems, Wilmington, NC), in which a constant bias flow supply connected to the animal recording chamber ensured continuous inflow of fresh air (1 l/minute). Only episodes of quiet breathing totaling at least 10 minutes were used for analysis. Quiet breathing was defined as periods when the animal was not moving and had all four paws on the chamber floor for a minimum of 2–3 minutes. Apneas were defined as breathing pauses longer than 2× the average duration of expiration (Te) measured during quiet breathing. Breathing traces were analyzed using Biosystem XA software (Buxco Research Systems, Wilmington, NC). Ambient temperature was maintained between 23 and 25°C. To define the acute effects of LM22A-4 on breathing, mice were given a single i.p. injection of either LM22A-4 (150 mg/kg body weight in saline) or an equivalent volume of saline alone (Control) 1 hour prior to plethysmography. During this 1-hour period, animals were left undisturbed to acclimate to the plethysmographic chamber and breathing measurements were then taken during the subsequent 3 hours. To define the time course of LM22A-4 effects on breathing, plethysmography was also performed 24 hours after injection in a subset of animals. LM22A-4 was purchased from Ricerca Biosciences, LLC (Concord, OH).

### Orienting reflex testing

Plethysmographic recordings were obtained as described above before, during and after exposure to a single 50-ms broad-spectrum auditory pulse (80 dB white noise). The acoustic stimulus was presented through two speakers (Advent) placed outside the plethysmographic chamber and the intensity level was calibrated using a sound meter (ANL-929A-PC; Med Associates, VT) placed inside the chamber. Animals were injected with saline or LM22A-4 as described above and reflex testing was performed once the animals exhibited at least 2 minutes of quite breathing (approximately 1 hour after being placed in the plethysmograph).

### Acoustic startle testing

The acoustic startle response was measured using a San Diego Instruments startle response recording system (San Diego, CA). Mice were placed inside a small-sized, non-restrictive, cylindrical Plexiglas recording chamber [3.5” (L)×1.1” (ID)] on an accelerometer platform. Following a 5-minute acclimation period inside the recording chamber with a constant 70 dB background white noise, a 40-ms white-noise stimuli of different intensities (70, 74, 78, 82, 86, 90, 100, 110 and 120 dB) were delivered four times each in a random order with random intervals. The maximum accelerometer response amplitude measured within 40 ms after the acoustic stimulus was defined as the acoustic startle response (*V*max). *V*max measurements obtained from each of the four repeated trials of the same stimulus intensity were averaged.

### Open-field testing

Locomotor activity was measured while the mouse was in an open field consisting of a 40×40 cm box located in a dimly lit room. Using EthoVision XT 5.0 software, the field was digitally subdivided into a 20×20 cm center area and a periphery. The periphery was further divided into middle (inner 10 cm) and outer (outer 10 cm) areas. 1 hour after injection with LM22A-4 or saline, animals were placed in the open field and allowed to explore the enclosure freely for 15 minutes. During this period we measured total distance moved, velocity, angular velocity, rearing and heading to determine basic locomotor activity, as well as frequency and duration in the center, periphery and outer areas to evaluate thigmotaxis. Additionally, data were nested into three 5-minute bins and the distance moved during each of these three periods was recorded to evaluate habituation differences across groups. Values for the middle and outer sections were added together to calculate the center:periphery ratio.

### Electrophysiology

#### Slice preparation

To study acute effects of LM22A-4 on synaptic transmission in the nTS, horizontal brainstem slices were prepared from 5- to 7-week-old Null and Wt male mice as previously described (Doyle and Andresen, 2001; Kline et al., 2002; Kline et al., 2010). In brief, mice were deeply anesthetized by inhalation of isoflurane and then decapitated. Brains were removed from the skull and placed for 2–5 minutes in ice-cold, low Ca^2+^ artificial cerebrospinal fluid (ACSF) containing (mM): NaCl, 125; KCl, 3; NaH_2_PO_4_, 1.2; CaCl_2_, 1; MgSO_4_, 1.2; MgCl_2_, 2; NaHCO_3_, 25; D-glucose, 10 and L-ascorbic acid 0.4, equilibrated to pH 7.4 with 95% O_2_/5% CO_2_. Brainstems were dissected, glued on the mounting platform of a vibratome (Leica, VT 1000S), and horizontal sections containing the nTS, including a long segment of the TS, were cut at 220–250 μm. Slices were then transferred to recording ACSF (mM: NaCl, 125; KCl, 3; NaH_2_PO_4_, 1.2; CaCl_2_, 2; MgSO_4_, 1.2; NaHCO_3_, 25; D-glucose, 10 and L-ascorbic acid, 0.4, equilibrated to pH 7.4 with 95% O_2_/5% CO_2_) at ~32°C and allowed to recover from the procedure for at least 30 minutes before recordings.

#### Recordings

Slices were placed into the recording chamber, held in place with a nylon-wired grid and superfused with recording ACSF at 30–32°C at a flow rate of 4–5 ml/minute. For stimulation of presynaptic inputs to nTS neurons, a concentric bipolar stimulation electrode (Frederic Haer) was placed on the TS, the medullary tract containing the central axons of primary afferent inputs to the nTS, rostral to recording sites. Patch pipettes were pulled from thick-walled borosilicate glass capillaries and filled with intracellular solution (mM: K+gluconate, 130; NaCl, 10; EGTA, 11; CaCl_2_, 1; HEPES, 10; MgCl_2_, 1; MgATP, 2; NaGTP, 0.2), and had resistances between 4 and 9 MΩ. Recordings were made lateral to the TS at the level of, and caudal to, the obex, which includes the interstitial, lateral and ventrolateral subnuclei [referred to as lateral nTS (lnTS)], the central targets of pulmonary stretch receptors (Kubin et al., 2006). Neurons were visualized with an upright Olympus microscope (BX51WIF). To mimic afferent sensory input, the TS was stimulated at 0.5 Hz. Only neurons receiving monosynaptic input on 0.5 Hz TS stimulation (20 sweeps), defined as a low jitter of latency of evoked postsynaptic responses (<250 μs), were considered. From these neurons, eEPSCs, sPSCs and mEPSCs were recorded in the whole-cell voltage-clamp configuration at a holding potential of −60 mV. To obtain lengthy eEPSC recordings during drug application, the TS was stimulated continuously at 0.1 Hz in these experiments. Neurons with a resting membrane potential more positive than −40 mV upon breakthrough were rejected. Series resistance (*R*_s_) was compensated (80%), and only neurons with stable *R*_s_ throughout the experiment were considered for the analysis of eEPSCs. Data were acquired using pClamp software. Signals were amplified (Axopatch 200B, Axon Instruments), filtered at 2 kHz and digitized at 10 kHz.

Control and drug recordings were performed in *n*=47 mice (*Mecp2^−/y^*) and *n*=30 mice (WT). For control measurements, one to four cells were recorded per slice. Because of potential long-lasting and non-reversible drug effects (LM22A-4, K252a), slices were not reused once exposed to a drug.

LM22A-4 effects on evoked and spontaneous synaptic currents in individual second-order lnTS neurons were recorded over a maximum of 20 minutes and compared with pre-drug baseline conditions in the absence and presence of the non-selective TrkB inhibitor K252a, using the drug exposure timeline described below.

### Data analysis

eEPSCs, sPSCs and mEPSCs were analyzed with Clampfit and Microsoft Excel. 0.1 Hz TS-stimulation-evoked EPSC amplitudes were analyzed during a 5-minute control interval and in segments of 5–10 minutes LM22A-4, 10–15 minutes LM22A-4 and 15–20 minutes LM22A-4, and respective time points in the presence of K252a. sPSC and mEPSC traces were digitally filtered at 1 kHz, and events were counted within 2-minute segments, during the following time points: control, 8–10 minutes LM22A-4, 13–15 minutes LM22A-4 and 18–20 minutes LM22A-4. Instantaneous frequencies and amplitudes were analyzed. The detection threshold for sPSCs and mEPSCs was set as 1–1.5× peak-to-peak noise.

### Statistical analysis

All data are presented as means±s.e.m. Experiments involving no more than two treatment groups were analyzed by unpaired two-tailed Student’s *t*-test. Multiple group data were analyzed by one-way ANOVA with post-hoc Tukey or least significant difference (LSD) tests for inter-group comparisons. Comparison of acoustic startle thresholds at multiple levels of startle intensity were analyzed by RM-ANOVA. For electrophysiological recordings, repeated measurements of drug effects on eEPSCs and sPSCs were analyzed with RM-ANOVA, followed by the Tukey post-hoc test. All mEPSCs recorded within the 2-minute control interval and within a 2- minute drug interval were pooled according to the genotypes, and frequency and amplitude distributions were compared with the Kolmogorov-Smirnov test. Cells that showed a percent change in EPSC amplitude (evoked or spontaneous) that was greater than two times the standard deviation were considered outliers and excluded from analysis (one cell each for eEPSC and sPSC data sets). Results were considered significant if the *P*-value was less than 0.05.

## Supplementary Material

Supplementary Material
