# Supplementary material for: A BDNF loop-domain mimetic acutely reverses spontaneous apneas and respiratory abnormalities during behavioral arousal in a mouse model of Rett syndrome
Source: Dis Model Mech. 2014 Sep;7(9):1047–55. doi: 10.1242/dmm.016030 (PMC4142725; doi:10.1242/dmm.016030)
Supplement: Supplementary Material [file supp_7_9_1047__index.html]

A BDNF loop-domain mimetic acutely reverses spontaneous apneas and respiratory abnormalities during behavioral arousal in a mouse model of Rett syndrome — Supplementary Material 

# A BDNF loop-domain mimetic acutely reverses spontaneous apneas and respiratory abnormalities during behavioral arousal in a mouse model of Rett syndrome

## DMM016030 Supplementary Material

**Files in this Data Supplement:**

- **Supplementary Material**
