# Supplementary material for: A BDNF loop-domain mimetic acutely reverses spontaneous apneas and respiratory abnormalities during behavioral arousal in a mouse model of Rett syndrome
Source: Dis Model Mech. 2014 Sep;7(9):1047–55. doi: 10.1242/dmm.016030 (PMC4142725; doi:10.1242/dmm.016030)
Supplement: Supplementary Material [file supp_7.9.1047_DMM016030.pdf]

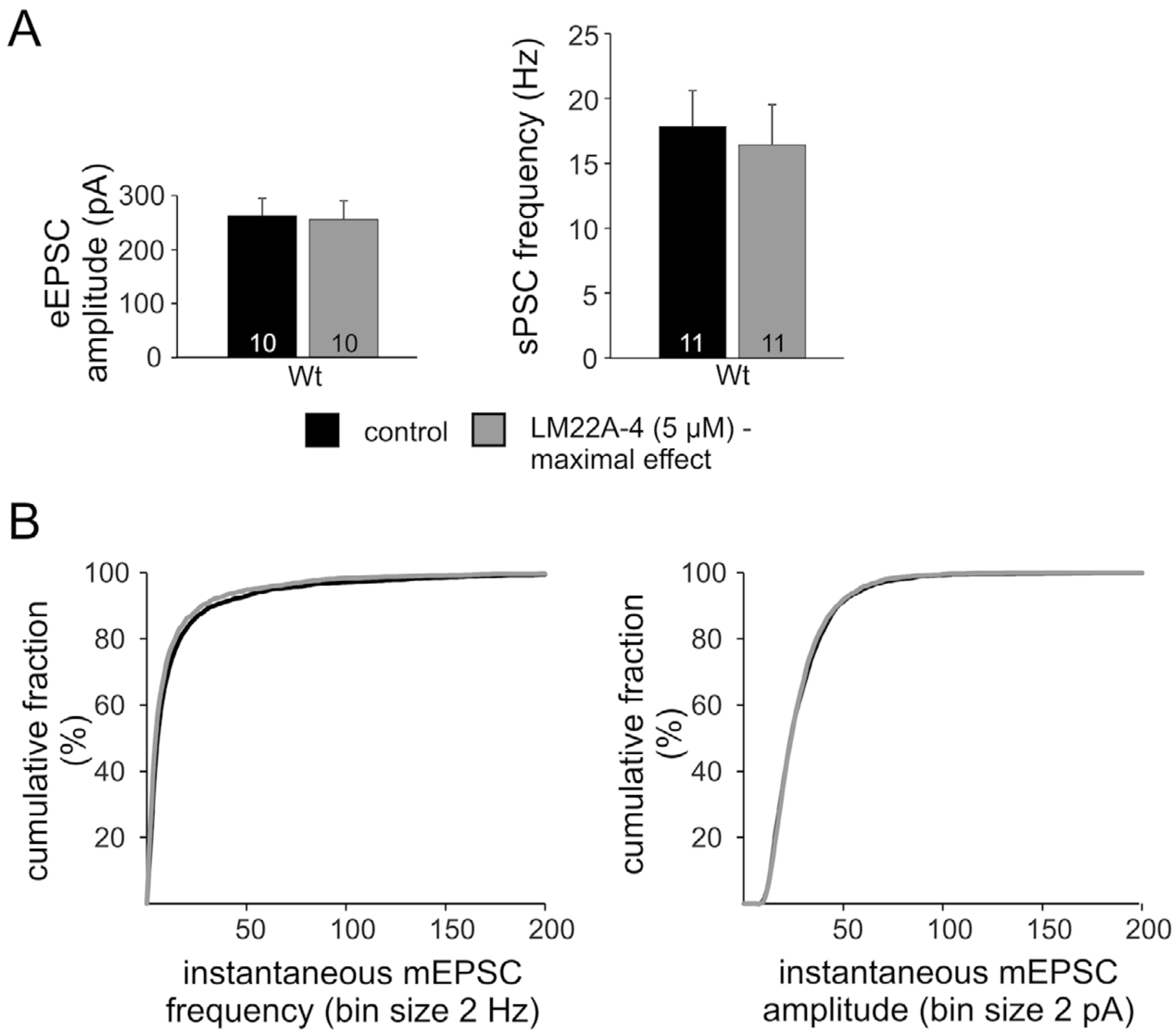

Fig. S1, Related to Fig. 2. LM22A-4 has small effects on synaptic function in Wt animals. (A) Summary graphs comparing the maximal effects of LM22A-4 on eEPSC amplitude and sPSC frequency in lnTS neurons from Wt mice. (B) Cumulative distribution plots of mEPSC frequency and amplitude recorded from Wt lnTS neurons in the absence (black) and presence (grey) of LM22A-4 reveal small left shifts in some regions that reach statistical significance (*P*<0.05, Kolmogorov–Smirnov test).

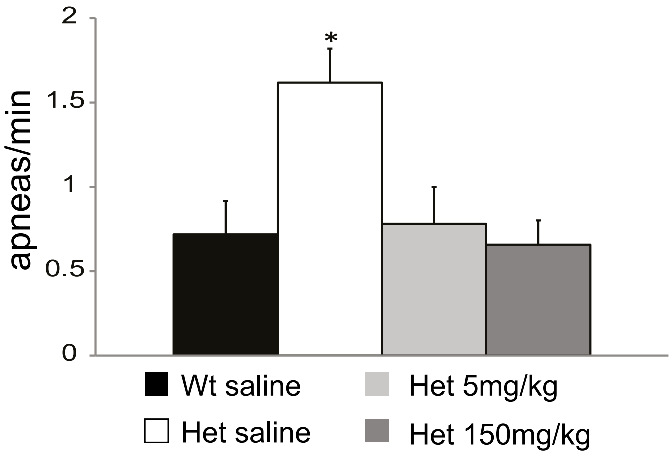

Fig, S2, Related to Fig. 4. Initial dose-ranging studies indicate that LM22A-4 is effective at reversing the apnea phenotype at 5 mg/kg. Summary graph showing acute reversal of the apneic phenotype in 16 week old Het mice following a single treatment with LM22A-4 at either 5 mg/kg or 150 mg/kg, i.p. one hour prior to the beginning of breathing data collection. *$P<0.05$ (ANOVA followed by LSD).
